# How regional innovation and entrepreneurship vitality affects residents’ leisure consumption potential—utility differences between educational investment and scientific and technological investment

**DOI:** 10.1371/journal.pone.0317742

**Published:** 2025-02-13

**Authors:** Yunying Cai, Wenhe Lin, Jinfa Zhong, Qiqi Hu, Yaqing You

**Affiliations:** 1 College of Economics and Management, Fujian Agriculture and Forestry University, Fuzhou, China; 2 College of Accounting, Yang-En University, Quanzhou, China; 3 College of Rural Revitalization, Fujian Agriculture and Forestry University, Fuzhou, China; Federal University of the ABC: Universidade Federal do ABC, BRAZIL

## Abstract

Innovation and entrepreneurship vitality, as a key factors in the development of the digital economy, significantly affects both regional economic development and residents’ consumption capacity. On the basis of the panel data of 31 provinces and cities in China from 2010 to 2022, this paper explores the impact of regional innovation and entrepreneurship vitality on residents’ leisure consumption potential and its internal mechanism. Research has shown that innovation and entrepreneurship vitality drives leisure consumption potential. Furthermore, regional innovation and entrepreneurship vitality can effectively increase educational investment, which in turn increases residents’ leisure consumption potential. Although scientific and technological investment can significantly increase innovation and entrepreneurship vitality, it curtails the development of residents’ leisure consumption potential. In addition, there are significant differences in the effect of regional innovation and entrepreneurial vitality on residents’ leisure consumption potential in different policy environments and demographic structure. Diverse policies help to better stimulate regional innovation and entrepreneurship influence on residents’ leisure consumption. The development of innovation and entrepreneurship vitality can effectively stimulate the leisure consumption potential of residents in areas with low population mobility, which compensate for the decrease of leisure consumption due to the lack of external population. On the basis of the inconsistency between economic development and residents’ spiritual needs in China’s major contradictions, this study further explores the coordination between scientific and technological development and individuals’ needs for a better life, and further complements and justifies the literature in the field of innovation and consumption.

## 1. Introduction

During the visit to Heilongjiang, General Secretary Xi Jinping proposed the integration of scientific and technological innovation resources to lead the development of strategic emerging industries and future industries [1]. Currently, technologization, digitization, and intelligence are violently sweeping the world and are important development trends for enterprises, societies, and countries. Driven by the wave of digitalization and intelligence, the concept of “innovation and entrepreneurship” has received a great deal of attention, and the lives of the population have changed considerably as a result of the development of the digital economy.

The main contradiction of China in the new era is that the people’s spiritual life needs are difficult to satisfy, and how to enhance the people’s sense of access, so that the masses can share the fruits of economic development is an important measure to meet the residents’ needs for a better life [2]. With the enhancement and increase in consumer demand, many enterprises enthusiastically carry out technological innovation and product breakthroughs, and guide the public to generate diversified consumption through diversified products and services. Among the various types of consumption, the pleasure generated by leisure consumption [3] is the most suitable for the spiritual needs of consumers, and has become a measure of the spiritual life of the public.In terms of the intrinsic connection between the economy and leisure, leisure scientist Ma Huidi argues that leisure is both an economic rewards and can be an effective economic participation in the form of consumption [4].

Currently, China’s leisure consumption suffers from structural irrationality and an imbalance between product supply and demand [5]; however, the wave of digitization is developing rapidly across the globe. The development of the digital economy has greatly stimulated the innovation and entrepreneurship vitality of the cities [6] and promoted the development of the social environment toward intelligence and convenience. The improvement of the living environment has greatly satisfied the material needs of the masses, allowing residents to start pursuing spiritual needs and leisure consumption. According to transaction cost theory, regional innovation and entrepreneurial vitality promotes consumption upgrading by improving the consumption environment, reducing information asymmetry, and increasing social welfare, thus enhancing residents’ potential for leisure consumption [7]. However, few in-depth studies have been conducted on the intrinsic influence mechanism between urban innovation and entrepreneurship and leisure consumption potential driven by digital technology.

On the basis of the global context of the rapid development of the digital economy, this study analyzes how each city influences the leisure consumption behavior of its residents in the process of improving innovation and entrepreneurship vitality. In addition, the relationship between government finance and the digital economy is complex. There is a two-way influence between the digital economy and government finance [8]. Based on the evolutionary game theory, this study further analyzes the effects of different kinds of government fiscal inputs on regional innovation and entrepreneurship and residents’ leisure consumption potential from three levels: enterprise, government and individual. The findings help to supplement the research gap between innovation and entrepreneurship vitality and leisure consumption potential, and helps to verify the incentive effect of urban science and technology development on residents’ deep-level consumption.The results of the study reinforce the assertion that “innovation is the first driving force for development” and promote the realization of synergistic development between the development of the digital economy and people’s spiritual needs in each country.

## 2. Theory and hypotheses

### 2.1. Theoretical foundation

#### 2.1.1. Evolutionary game theory.

Evolutionary game theory aims to explain the strategic choices of participating subjects under the state of incomplete information and limited rationality, and to explain the dynamic game process of how different subjects can reach an equilibrium and stable state [9]. Evolutionary games are characterized by the ability to form a dynamic analytical framework from multiple theoretical perspectives to analyze complex problems with the participation of multiple players [10]. Therefore, evolutionary game theory has been widely used in the social sciences, behavioral sciences, and economic and management fields [11].

Based on the evolutionary game theory, this study analyzes the effects of government finance, corporate innovation capacity and individual leisure consumption potential from three levels: corporate, government and consumer. It has been shown that government subsidies and special funds are important incentives to promote business innovation, digital transformation and residents consumption [12]. Digital innovation in business promotes more convenient and diversified consumption and is a reflection of the efficient transformation of government finances [13]. Leisure consumption helps to increase government revenues and stimulates innovative and creative behavior in enterprises [14]. Therefore, in the context of the digital economy, there is a dynamic mutual influence relationship between enterprises, governments and consumers. By analyzing the dynamic behavioral decision-making among different subjects, it helps to enhance the relevance of the research problem and the realistic guidance of the research conclusions.

#### 2.1.2. Transaction cost theory.

Transaction cost theory suggests that opportunism in the transaction process is an important factor in whether transaction costs are incurred or not, and that information asymmetry and earmarked investment are important factors affecting opportunism [15,16]. According to the transaction cost theory, the level of regional innovation is a key factor affecting transaction costs, and the results of regional innovation influence trading behavior. In the context of the digital economy, innovative and entrepreneurial behavior helps to improve the information transparency of the trading market, reduce transaction costs, further monitor and safeguard the interests of both parties to the transaction [17], and better promote the trading partnership. To promote the prosperity and growth of the trading market, the government or enterprises will be more active in investing in innovative and entrepreneurial behavior, thus enhancing the innovation and entrepreneurship vitality of the whole city. The innovation and entrepreneurship vitality of a city contributes to the efficient integration of existing resources, facilitates the process of information sharing [18], increases consumer confidence [19], and promotes transactional behavior.

**Fig 1:**
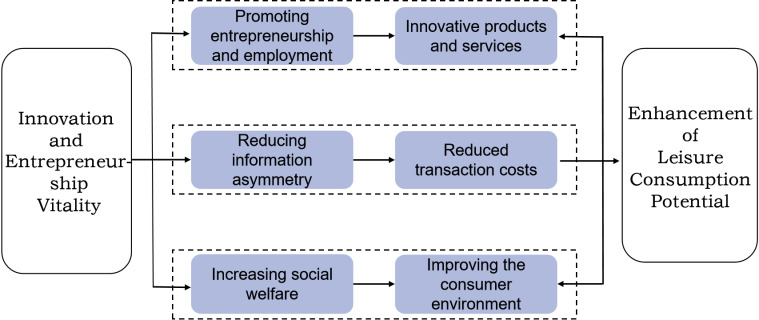
Mechanism of regional innovation and entrepreneurship vitality on leisure consumption potential.

In summary, the innovation and entrepreneurship vitality mainly reduces the transaction costs of consumers by amplifying the innovation advantage, weakening the information disadvantage and enhancing the consumption guarantee, thus further stimulating the leisure consumption potential of residents(see Fig 1 for details). Based on transaction cost theory and evolutionary game theory, this study explores how innovation and entrepreneurship dynamism in each region of China affects residents’ leisure consumption potential at the levels of firms, governments and consumers. In addition, the study further examines whether scientific and technological inputs and educational inputs in each region play a role in the trading process, and analyzes in depth how various types of government fiscal expenditures affect opportunism during the trading process.

### 2.2. The impact of regional innovation and entrepreneurship vitality on residents’ leisure consumption potential

Innovation and entrepreneurship vitality refers to the degree of development of new enterprises, new capital and new technologies, which is an important manifestation of new quality productive forces [20], and an important manifestation of the digital economy empowering high-quality development [21]. Among the factors affecting innovation and entrepreneurship vitality, the emergence of digital technologies, digital platforms, and digital infrastructures greatly influences innovation and entrepreneurship, providing new opportunities for innovators and entrepreneurs [22], and realizing entrepreneurial goals by creating a favorable external environment [23].

Leisure consumption refers to the consumption activities of leisure products and services in people’s leisure time, and it is the experiential consumption of culture, entertainment, fitness, tourism, and tournaments in addition to daily consumption such as clothing, food, housing and transportation [24]. It also encompasses consumption activities that satisfy personal developmental as well as spiritual needs, such as knowledge and skill acquisition activities [25]. Leisure consumption is considered to be hedonic consumption [3], reflecting the degree of satisfaction of people’s needs for a better life [26].

Leisure consumption potential is a comprehensive reflection of residents’ leisure consumption demand and potential consumption willingness, and scholars have analyzed the characteristics and influencing factors of leisure consumption from the perspectives of economics and sociology [27]. The factors influencing residents’ leisure consumption potential can be divided into two aspects: from the objective level containing factors such as income level, leisure time, the social environment, the cost of living, large-scale events [28], and hot trends, and subjective factors, such subjective well-being [29], social space [30], consumption habits, consumption concepts, consumption culture, the psychological state and other factors. The level of leisure supply is the necessary condition and basic threshold of leisure consumption [31], whereas innovation and entrepreneurship vitality affects the level of supply and determines the upper limit of the potential of leisure consumption to a certain extent.

Studies have validated the ability to innovate, and digital technology has helped to drive social consumption upgrades. Innovation and entrepreneurship vitality can participate in the consumption process of the population in the form of digital technology and digital finance. Studies have shown that digital payment technology helps to increase the convenience of consumption and thus the level of consumption of the population [32]. Digital finance helps residents gain additional wealth effects, helping them to break through consumption constraints and bring a sense of financial security, thus increasing households’ current consumption capacity and willingness to consume [33]. In addition, scholars have shown that the application of digital technologies, such as big data, has fundamentally changed the traditional concept of residents’ consumption and significantly improved the quality of residents’ consumption, thus promoting consumption upgrading [34].

In the context of transaction cost theory and evolutionary game theory, regional innovation and entrepreneurship vitality can increase consumption demand through three aspects: promoting innovative employment, reducing information asymmetry, and increasing social welfare, thus promoting leisure consumption potential [35]. The ability to innovate and digital technology can help improve information asymmetries and increase the frequency of individual consumption, spending [36] and the desire to pursue deeper consumption [37]. Science and technology innovations enable consumers to meet their changing needs and help upgrade and evolve the consumption structure [38]. Therefore, leisure consumption, as a deep-level consumption of residents to satisfy spiritual needs, is closely related to the level of social innovation and development. This study proposes the following hypotheses:

H1: Regional innovation and entrepreneurship vitality will contribute to enhancing residents’ leisure consumption potential.

### 2.3. Mediating effect of education investment

According to transaction cost theory, earmarked investment significantly enhances collaborative innovation performance [39,40], and government finance serves as an important source of investment in the development of urban innovation performance. Studies have shown that fiscal spending has a two-way effect. Some scholars believe that fiscal spending is direct government consumption and that increases government spending will increase total social output or total income [41,42], which can promote the level of consumption of residents and households; at this time, government finances are manifested in the “crowding-in effect” on consumption. There are also scholars who believe that government fiscal expenditure will trigger inflation, increase the price level, and increase the pressure on households to consume, resulting in fiscal expenditures manifesting themselves as a “crowding out effect” on residents’ consumption [43]. Although existing studies have not yet harmonized whether fiscal spending is a “crowding-in effect” or a “crowding-out effect”, China’s finance has strong attributes, and it can be adjusted continuously to make fiscal spending most beneficial to the people. Therefore, the role played by the government’s fiscal expenditure between regional innovation and entrepreneurship and residents’ consumption deserves to be further explored.

Currently, China’s fiscal expenditure includes educational fiscal expenditure, science and technology fiscal expenditure, social welfare expenditure, etc. Constructing a good complete and fair innovation fiscal mechanism [44], and rationally carrying out the distribution of fiscal expenditure are particularly important for developing regional innovation and entrepreneurship vitality. In terms of the relationship between fiscal expenditure and increased consumption, scholars have noted that fiscal spending should be directed toward supporting innovation to meet the new round of domestic demand expansion strategies [45]. In the process of innovation and entrepreneurship education and the cultivation of innovative talent, education investment plays an important role [46], so education financial investment is an important element of financial investment in the current innovation trend.

Fiscal expenditure on education plays a crucial role. On the one hand, it has a direct effect on the effectiveness of the training of high-level human resources; on the other hand, it determines the cost of education to be borne by individual families and affects their leisure consumption potential. Research has shown that innovation drives the economy, which is closely linked to higher education rates [47]. Existing studies have focused mainly on how educational investment influences innovation and entrepreneurship, and have investigated the different uses and effects of educational investment. Educational investment promotes the interface between innovation and entrepreneurship education and economic development in higher education by upgrading teaching conditions, increasing faculty, developing innovation and entrepreneurship education, and building university platforms [48], thus stimulating and driving diversified consumption. The impact between regional innovation and entrepreneurship vitality and educational investment is bidirectional, high investment in education contributes to better innovation and entrepreneurship vitality [49], and high innovation and entrepreneurship vitality effectively contributes to the growth of educational investment and reduces the financial burden on households, thereby increasing their leisure consumption potential. Thus, this study proposes the following hypothesis:

H2: There is a mediating effect of educational investment between regional innovation and entrepreneurship vitality and residents’ leisure consumption potential.

### 2.4. Moderating effect of scientific and technological investment

Scientific and technological investment is an effective means to realize the synergistic development of economic growth and technological progress [50]. In the process of increasing the level of regional innovation and entrepreneurship, strengthening fiscal expenditure on science and technology is a direct and effective way [51]. Currently, innovation incentives are used by governments around the world to guide and promote local enthusiasm for technological innovation, which is manifested in the year-to-year increase in fiscal expenditure on science and technology.

Research has shown that fiscal expenditure on science and technology can alleviate the financing constraints of enterprises, stabilize their financial status, and optimize their innovative behavior at the level of “input-output” [52]. In addition, fiscal expenditure on science and technology can accelerate the construction of local innovation systems, and actively guide and support the innovation and entrepreneurship activities of local enterprises, colleges and universities, and scientific research institutions, to promote the development of local innovation [53]. Science and technology fiscal expenditure helps to stimulate scientific and technological innovation, and the introduction of good scientific research projects help develop the regional economy; however, recently, many regional governments’ science and technology fiscal expenditure have not received the expected feedback from the market, and science and technology fiscal expenditure has led to distorted behavior [54].

In recent years, China’s macroeconomy has faced greater downward pressure due to repeated disturbances from the new crown epidemic, a complex and volatile geopolitical situation, and tightening monetary policies in major economies [8]. Studies have shown that the use of financial resources from other areas for digital innovation in the context of fiscal pressures is not conducive to the coordinated development of the digital economy in all regions [55]. In particular, the rapid promotion of digital industrialization and digitization of industries and the cultivation of new quality productivity may result in duplication of investment, over-investment and irrational investment structures [56].

According to existing research, the negative effect of scientific and technological investment on innovation development and leisure consumption is manifested in two main ways. On the one hand, under the stimulation of science and technology policy, there is competition between science and technology in different places, which induces many enterprises to carry out a large amount of research and development in isolation from the social reality and market demand, resulting in “the waste of scientific research” that is not beneficial to the operation of the market and the development of society [57]. On the other hand, as each region continues to increase its fiscal expenditure on science and technology, it has led to a relative decline in the fiscal expenditure for people, which is directly related to the individual residents, a reduction in the social welfare of the residents, and the occurrence of fiscal competition between technology and the masses.Therefore, this paper proposes the following hypothesis:

H3: There is a moderating effect of scientific and technological investment between regional innovation and entrepreneurship vitality and residents’ leisure consumption potential.

## 3. Method

### 3.1. Sample selection and data sources

The purpose of this paper is to study the impact of innovation and entrepreneurship levels on leisure consumption ability in different regions of China. Therefore, 31 provinces and cities in China with relatively sound and standardized statistical indicators of various types from 2010--2022 are selected as the research samples, and the missing values and outliers in the samples are eliminated.

### 3.2. Variables and measures

#### 3.2.1. Dependent variable: leisure consumption potential.

Leisure consumption potential is the manifestation of residents’ real and potential leisure consumption demand [31]. Leisure consumption potential has rich connotations and various influencing factors. The consumer economist Yin Shijie has analyzed the influencing factors of leisure consumption potential in a more comprehensive way, in which the influence of objective factors such as residents’ income level, the consumption environment, social welfare and leisure consumption goods is more significant [58]. Therefore, this study focuses on analyzing the objective factors affecting leisure consumption. According to the research of Lou Jiajun [59] and other scholars to measure the leisure consumption potential of residents in different regions with three indicators: the consumer price index, per capita disposable income, and per capita consumption expenditure of residents’ households. In the measurement of the weights of the indicators, the method of combining the subjective judgment method and the objective analysis method is adopted, i.e., 50% of the weight of the subjective judgment method plus 50% of the weight of the objective analysis method is added to form the weights of each indicator in evaluating the leisure consumption potential.

(1) Subjective judgment method weighting

$$\lambda_{j}^{a}=\bar{X\text{j}}/\sum_{j=1}^{3}\bar{X}\;\left(i=1,2,3......31,j=1,2,3\right)$$ (1)

(2) Objective analysis method weighting

The coefficient of variation of the indicator variable series is as follows:


$$Vj=Sj/\bar{X\text{j}}$$ (2)

$$\bar{X\text{j}}=\sum_{i=1}^{31}Xij/31,\;Sj=\sqrt{\frac{1}{31}\sum_{i=1}^{31}{\left(Xij-\bar{Xj}\right)}^{2}}$$ (3)

$$Weigh:\;\lambda_{j}^{b}=Vj/\sum_{j=1}^{3}Vj$$ (4)

The weight of the evaluation system variables is as follows:


$$\lambda j=(\lambda_{j}^{a}+\lambda_{j}^{b})/2$$ (5)

#### 3.2.2. Independent variable: innovation and entrepreneurship vitality.

This study measures the innovation and entrepreneurship vitality of the regions with the new quality productivity index. New quality productivity is defined as productivity in which science and technology innovation plays a leading role [60], which helps to realize high-level scientific and technological self-reliance and promotes the realization of high-quality development of China’s economy in the new era [61]. In this study, we refer to the study of Lu Jiang and other scholars [62] to measure and count the new quality productivity index in each region. Through a comprehensive system of three first-level indicators (scientific and technological productivity, green productivity and digital productivity), six second-level indicators and 18 third-level indicators, the improved entropy weight TOPSIS method was used to assign weights to the indicators, so as to obtain the development level of new quality productivity in each region of China (the details are shown in Table 1).

**Table 1:** Indicator system for evaluating new quality productivity.

First Level	Second Level	Third Level	Interpretation	Attribute
Technological Productivity	Innovative productivity	Innovative R&D	No. of domestic patents granted	+
		Innovative industry	Business income from high-tech industry	+
		Innovative products	Industrial innovation funding for standard-sized enterprises	+
	Technological productivity	Technical efficiency	Labor productivity of standard-sized enterprises	+
		Technical R&D	Full-time equivalents of R&D personnel of standard-sized enterprises	+
		Technical production	Robot mounted raw density	+
Green Productivity	Resource- efficient productivity	Energy intensity	Energy consumption/GDP	–
		Energy structure	Fossil energy consumption/GDP	–
		Water intensity	Industrial water use/GDP	–
	Environmen- tally friendly productivity	Utilization of waste	Comprehensive utilization/generation of industrial solid waste	+
		Wastewater discharge	Industrial wastewater discharges/GDP	–
		Exhaust emission	Industrial SO_2_ emissions/GDP	–
Digital Productivity	Digital industry productivity	Electronic information manufacturing	IC production	+
		E-business newsletter	Total telecommunications business	+
	Industry digital productivity	Internet penetration	Internet broadband access ports	+
		Software service	Software business income	+
		Digital information	Length of fiber optic lines/area	+
		E-commerce	E-commerce sales	+

(1) Normalization of indicators

Considering that the entropy weighting method used below requires the indicator value to be greater than zero, this paper adopts the extreme value processing method, so that the data after the indicator normalization process will finally fall within the interval of [0, 1], the specific formula is as follows:


$$x_{ij}^{*}=\frac{x_{ij}-m_{j}}{M_{j}-m_{j}}$$ (6)

Among them,$M_{j}=\underset{i}{\max}\left\{x_{ij}\right\},m_{j}=\underset{i}{\min}\left\{x_{ij}\right\}$

(2) Calculation of entropy weight method coefficient of variation

The entropy weight method assigns specific weights to the corresponding indicators based on the amount of information that each indicator can provide. According to the traditional entropy value method, the coefficient of variation needs to be calculated first, and the specific calculation method is as follows:

Calculate the characteristic weight p_ij_ of the ith evaluated object under the jth indicator.


$$p_{ij}=\frac{x_{ij}}{\sum_{i=1}^{m}x_{ij}}$$ (7)

Among them, m is the number of samples.

Calculate the entropy value e_j_ for the jth term.


ej=1lnm∑i=1mpijln1pij (8)

Among them, $I_{ij}=\ln\frac{1}{p_{ij}}$denotes the amount of information.

$I_{j}=\sum_{i=1}^{m}p_{ij}\ln\frac{1}{p_{ij}}$denotes the total amount of information and ej is the entropy value.

Calculate the coefficient of variation g_j_ for the jth indicator.


$$g_{j}=1-e_{j}$$ (9)

(3) Calculate the mapping value in the improved entropy weight method

The improved entropy weight method follows the basic idea of AHP, first compare the difference coefficients of the traditional entropy weight method, map the result to the 1-9 scale of AHP, and then get the pairwise comparison matrix based on entropy weight, and then get the weights by normalizing the matrix according to the basic operation of AHP, and its specific steps are as follows:

Calculate the maximum coefficient of variation ratio D.


$$D=\frac{\max g_{j}}{\min g_{j}}$$ (10)

Calculate the mapping ratio R for the 1-9 scale.


$$R=\sqrt[{\text{a}-1}]{\frac{D}{a}}$$ (11)

Among them, a is the adjustment factor. If a ≤  9, then a takes the integer closest to D. If a >  9 then a takes 9 [63].

Calculate the mapping values of the criteria.

The mapping values required to improve the entropy weighting method can be calculated based on the 1-9 scale in AHP, and the corresponding principles are shown in Table 2.

**Table 2:** Hierarchical analysis scale mapping values.

Order	1	2	3	4	5	6	7	8	9
*RI*	1 * R0	2 * R1	3 * R2	4 * R3	5 * R4	6 * R5	7 * R6	8 * R7	9 * R8

Construct a judgment matrix to solve for the weights.

Calculate the ratio of the coefficient of variability between two pairs of indicators with the formula$r_{jk}=\frac{g_{j}}{g_{k}}$.

The closest value to r in the RI of Table 2 is taken to construct the pairwise judgment matrix, and then the hierarchical ranking and test are carried out according to the basic principles of the AHP method to obtain the final weights.

(4) Evaluation using the approximation of ideal points method

The approximation of ideal points method, or TOPSIS method, is a widely used objective and comprehensive evaluation method that highlights the overall differences, which emphasizes the principles of program discernment and free competition [64], and can achieve complementarity with the advantages of entropy weight method and other methods.

Construct a weighted normalization matrix based on the weights determined by the entropy weighting method.


$$V=P\times W=\left[\begin{array}{cccc} w_{1}P_{11} & w_{2}P_{21} & \cdots & w_{n}P_{m1}\\ w_{1}P_{12} & w_{2}P_{22} & \cdots & w_{n}P_{m2}\\ \vdots & \vdots & \cdots & w_{n}P_{m3}\\ w_{1}P_{1n} & w_{2}P_{2n} & \cdots & w_{n}P_{mn} \end{array}\right]$$ (12)

Calculate the optimal and worst case scenarios.

For the positive indicator, the optimal and worst solutions are respectively:


$$V^{+}=\left\{{\text{max}}_{j}V_{ij}|i=1,2\cdots n\right\}�V^{-}=\left\{{\text{min}}_{j}V_{ij}|i=1,2\cdots n\right\}$$ (13)

For the negative indicators, the optimal and worst solutions are, respectively:


$$V^{+}=\left\{{\text{min}}_{j}V_{ij}|i=1,2\cdots n\right\}�V^{-}=\left\{{\text{max}}_{j}V_{ij}|i=1,2\cdots n\right\}$$ (14)

Calculate the distance of the indicator from the optimal and worst options.


$$d_{i}^{+}={\left[\sum_{j=1}^{n}{\left(V_{ij}-V_{j}^{+}\right)}^{2}\right]}^{\frac{1}{2}}�d_{i}^{-}={\left[\sum_{j=1}^{n}{\left(V_{ij}-V_{j}^{-}\right)}^{2}\right]}^{\frac{1}{2}}$$ (15)

Among them, i = 1,2... m.

Calculate the relative fit.


$$c_{i}=\frac{d_{i}^{-}}{d_{i}^{+}+d_{i}^{-}}$$ (16)

The relative fit c_i_ reflects the closeness of the evaluation object to the optimal solution, which is the final score.

#### 3.2.3. Mediator and moderator.

On the basis of the different utilities of fiscal expenditure, through the literature, education investment is used as the mediating variable of the study, and the fiscal expenditure on education of each province is used as the measure of educational investment in each region; scientific and technological investment is used as the moderating variable of the study, and the fiscal expenditure on science and technology of each province is used as the measure of scientific and technological investment in the region.

#### 3.2.4. Control variables.

The social dimension may influence the results of the study.The following variables were controlled for: macroeconomic structure (MS), average years of schooling (AYS), total length of lines operated by public buses and trams (PBT), daily urban sewage treatment capacity (STC), combined population coverage of television programs (TV) and import volume(IMP). We also controlled for year and region dummy variables.

### 3.3. Model construction

#### 3.3.1. Main effect test.

To test the impact of innovation and entrepreneurship vitality on residents’ leisure consumption, the two-way fixed effect panel model of regional innovation and entrepreneurship vitality on residents’ leisure consumption potential constructed in this paper is shown in (17):


$${\text{LCP}}_{\text{i,t}}=\alpha_{\text{0}}+\alpha_{\text{1}}{\text{IEV}}_{\text{i,t}}+\alpha_{\text{2}}{\text{X}}_{\text{i,t}}+\lambda_{\text{i}}+\gamma_{t}+\varepsilon_{i,t}$$ (17)

LCP_i,t_ denotes the leisure consumption index of province i in year t; IEV_i,t_ represents the innovation and entrepreneurship vitality index of province i in year t; X_i,t_ is a series of control variables;*λ*_*i*_ and *γ*_*t*_ are the area and time fixed effects, respectively; and *ε*_*i,t*_ is the random error term.

#### 3.3.2. Mediation effect test.

To test whether educational investment plays a mediating role between innovation and entrepreneurship vitality and residents’ leisure consumption, this paper refers to the stepwise regression method of summarized by Wen [65] to construct a mediating effect model, which is shown in (18) and (19).


$${\text{EI}}_{\text{i,t}}=\beta_{\text{0}}+\beta_{\text{1}}{\text{IEV}}_{\text{i,t}}+\beta_{\text{2}}{\text{X}}_{\text{i,t}}+\lambda_{\text{i}}+\gamma_{t}+\varepsilon_{i,t}$$ (18)

LCPi,t=γ0+γ1IEVi,t+γ2EIi,t+γ3Xi,t+λ+iγt+εi,t (19)

EI_i,t_ denotes the educational fiscal expenditure in province i in year t.

In the first step, the significance of *α*_*1*_ is tested. If *α*_*1*_ is significant, it indicates that there is an overall effect between innovation and entrepreneurship vitality and residents’ leisure consumption potential, and we can continue the second step of mediation effect analysis;if *α*_*1*_ is not significant, it indicates that there is no significant overall effect between innovation and entrepreneurship vitality and residents’ leisure consumption potential, and then, we stand according to the masking effect and stop the analysis of the mediation effect. The second step is to test the significance of *β*_*1*_ and *γ*_*2*_. If both *β*_*1*_ and *γ*_*2*_ are significant, it indicates that educational investment plays a mediating role between innovation and entrepreneurship vitality and residents’ leisure consumption and continues to the third step; if at least one of *β*_*1*_ or *γ*_*2*_ is not significant, then the fourth step bootstrap test is performed.

The third step is to test the significance of *γ*_*1*_. If *γ*_*1*_ is significant, it indicates that there is a partial mediating effect of educational investment in the process of innovation and entrepreneurship vitality and residents’ leisure consumption, and the mediating effect is *β*_*1*_×*γ*_*2*_*/γ*_*1*_; if *γ*_*1*_ is not significant, it indicates that there is a complete mediating effect of education investment in the process of innovation and entrepreneurship vitality and residents’ leisure consumption. In the fourth step, a bootstrap test is conducted. If it passes the significance test, it indicates that the mediating effect of educational investment on the influence process of innovation and entrepreneurship vitality and residents’ leisure consumption; if it does not pass, it indicates that there is no mediating effect of educational investment on the influence process of innovation and entrepreneurship vitality and residents’ leisure consumption.

All relative variables are shown in Table 3.

**Table 3:** Description of variables.

Variable Type	Variable Symbol	Variable Name	Variable Measure
**Dependent** **Variable**	LCP	Leisure Consumption Potential	Subjective Judgment and Objective Analysis Method
**Independent Variable**	IEV	Innovation and Entrepreneurship Vitality	Entropy Weight TOPSIS Measures
**Mediating** **Variable**	EI	Educational Investment	National Bureau of Statistics Data
**Moderating** **Variable**	STI	Scientific and Technological Investment	
**Heterogeneous** **Variable**	EP	Structure of the External Population	Extra Provincial Population/Provincial Household Population
**Instrumental** **Variable**	IP	Innovation Performance	Evaluation Report of China’s Regional Innovation Capability
**Control Variables**	MS	Macroeconomic Structure	Sum of Value Added of Secondary and Tertiary/GDP
	AYS	Average Years of Schooling	National Bureau of Statistics Data
		PBT	Total Length of Public Buses and Tram Lines
		STC	Daily Urban Sewage Treatment Capacity
		TV	Comprehensive Population Coverage of TV Programs
		IMP	Import Volume
	YEAR	Year	Year Dummy Variable
	PRO	Province	Province Dummy Variable

## 4. Empirical analysis

### 4.1. Descriptive statistics

Table 4 presents the descriptive statistics of the main variables. The number of valid samples is 403. From 2010--2022, the mean value of the leisure consumption potential index of each region in China is 44.0111, the standard deviation was 17.4578, the minimum value was 19.85, and the maximum value was 100, which indicates that there are some differences in the leisure consumption potential of each region. The mean value of the innovation and entrepreneurship vitality index of each region is 0.1937, the standard deviation is 0.1766, the minimum value is 0.0267, and the maximum value is 0.8768, indicating that there are significant differences in the level of innovation and entrepreneurship vitality in different regions. Overall, the standard deviation of each variable is within a reasonable range, basically excluding the possibility of outliers interfering with the accuracy of the test results.

**Table 4:** Descriptive statistics of the main variables.

Variable	Number of samples	Mean	SD	Minimum	Maximum
**LCP**	403	44.01	17.46	19.85	100
**IEV**	403	0.19	0.18	0.03	0.88
**EI**	403	848.84	591.38	60.8	3871.14
**STI**	403	132.14	171.80	2.71	1168.79
**AYS**	403	9.16	1.14	4.22	12.68
**MS**	403	0.87	0.11	0.57	1.10
**PBT**	403	27484.52	28988.21	790	187331
**STC**	403	551.19	486.856	5	2971.30
**TV**	403	98.71	1.41	91.4	100
**IMP**	403	6.36e + 07	1.04e + 08	40000	4.97e + 08

### 4.2. Correlation analysis

Prior to the regression analysis, the Pearson test was performed on the correlated variables. The results of the Pearson test are shown in Table 5 Most of the results show that most of the variables are significantly correlated at the 1% level, and the correlation coefficients between the variables generally do not exceed 0.7, indicating that the sample data are reasonably selected for regression analysis. The correlation coefficient between the control variable average years of schooling(AYS), import volume(IMP) and the dependent variable leisure consumption potential (LCP) is greater than 0.7, which may be due to the correlation between the indicator systems measuring the two. The correlation coefficient between the moderator variable science and technology investment (STI) and the mediator variable education investment (EI) is greater than 0.7, which may be due to the correlation between the two indicator measures. To test whether there is a collinearity problem between the variables, the variables were tested for multicollinearity (Table 6). The variance inflation factors for each variable are much less than 10, indicating that there is no serious problem of multicollinearity, and regression analysis can be carried out to further study the relationship between the variables.

**Table 5:** Pearson test.

Variable	LCP	IEV	EI	STI	MS	AYS	PBT	STC	TV	IMP
**LCP**	1.000									
**IEV**	0.61***	1.000								
**EI**	0.28***	0.75***	1.000							
**STI**	0.58***	0.84***	0.83***	1.000						
**MS**	0.43***	0.45***	0.34***	0.44***	1.000					
**AYS**	0.74***	0.39***	0.26***	0.41***	0.17***	1.000				
**PBT**	0.34***	0.71***	0.85***	0.74***	0.27***	0.26***	1.000			
**STC**	0.41***	0.86***	0.86***	0.83***	0.28***	0.32***	0.81***	1.000		
**TV**	0.53***	0.41***	0.41***	0.43***	0.28***	0.62***	0.45***	0.41***	1.000	
**IMP**	0.77***	0.86***	0.58***	0.79***	0.45***	0.53***	0.58***	0.73***	0.39***	1.000

Note: ***, **, and *  represent significant at the 1%, 5%, and 10% levels, respectively

**Table 6:** Multicollinearity test.

Variable	STC	IEV	EI	IMP	STI	PBT	AYS	TV	MS	Mean VIF
**VIF**	7.90	7.60	5.00	4.52	2.59	2.28	2.05	1.51	1.55	3.84
**1/VIF**	0.13	0.13	0.14	0.17	0.17	0.25	0.45	0.50	0.65	

### 4.3. Benchmark regression analysis

On the basis of the issue of panel data model selection, first, the F test is used for fixed effect model and mixed regression model selection, and the test results show that the p value is 0.0000, which rejects the original hypothesis that there is no difference in the constant term of each cross-section; therefore, the fixed effect model is more matched than the mixed model. Second, regarding the selection of the fixed effect model and random effect model, this paper conducts the Hausman test and the p value is 0.0000; the original hypothesis that the random effect model is more effective is rejected, so it is reasonable to use the fixed effect model.

Table 7 shows the regression results of innovation and entrepreneurship vitality on the leisure consumption potential. The regression coefficient for regional innovation and entrepreneurship dynamics is 5.16 and significant at the 5% level (t = 2.13,p = 0.041). The regression results indicate that the innovation and entrepreneurship vitality of the region promotes residents leisure consumption potential. Test Hypothesis 1.

**Table 7:** Impact of innovation and entrepreneurship vitality on leisure consumption potential.

Variable	NQP	MS	AYS	PBT	STC	TV	IMP	YEAR	PRO	N	R^2^
**LCP**	5.16** (2.42)	−***9.98 (2.24)	1.63** (0.62)	0.00*** (0.00)	0.00 (0.00)	0.76*** (0.19)	−0.00 (0.00)	control	control	403	0.87

Note: ***, **, and *  represent significant at the 1%, 5%, and 10% levels, respectively

### 4.4. Robustness check

To verify the robustness of the regression results, the measure of replacing the explanatory variable innovation and entrepreneurship vitality index is used to test the regression results. The study uses the 2010-2020 China Regional Innovation and Entrepreneurship Index (IRIEC) compiled by Peking University’s Enterprise Big Data Research Center to represent regional innovation and entrepreneurship vitality. IRIEC is based on the three core elements of entrepreneurs, capital and technology.

The China regional innovation and entrepreneurship index (IRIEC) integrates the national industrial and commercial enterprise registration database, the VCPE investment database, the patent database, and the trademark registration database. A total of eight indicators in six dimensions, including the number of new enterprises (20%), attracting inward investment (15%), attracting venture capital (25%), the number of patents granted (20%), the number of trademarks registered (10%), and the number of software copyright registrations (10%), were used to assess the performance of innovation and entrepreneurship in the digital economy in each region [66]. As can be seen in Table 8, the coefficients of the explanatory variables are significantly positive after replacing the explanatory variable measures, which is consistent with the results of the benchmark regression, indicating that the results are robust.

**Table 8:** Robustness test after changing the explanatory variable measures.

Variable	IRIEC	MS	AYS	PBT	STC	TV	IMP	YEAR	PRO	N	R^2^
**LCP**	0.03** (0.01)	-7.26*** (2.12)	0.93 (0.55)	0.00*** (0.00	0.00 (0.00)	0.57*** (0.14)	-0.00 (0.00)	control	control	341	0.86

Note: Cluster robust standard errors are in parentheses; ***, **, and *  represent significance at the 1%, 5%, and 10% levels, respectively.

### 4.5. Endogeneity test

#### 4.5.1. Variable lag method.

To control the endogeneity problem between regional innovation and entrepreneurship vitality and residents’ leisure consumption potential as much as possible, this paper refers to the practice of Tang [66], which lags the core explanatory variables by one period and two periods to eliminate the problem of endogeneity caused by the reverse causality of “The higher the resident’ leisure consumption potential is, the greater the innovation and entrepreneurship vitality of the region”. As shown in Table 9, row (1) shows the results of the explanatory variable innovative entrepreneurial vitality (IEV) lagged 1 period and row (2) shows the results of the explanatory variable innovation and entrepreneurship (LCP) vitality lagged 2 periods.

**Table 9:** Endogeneity test for variable lags.

Variable	IEV	MS	AYS	PBT	STC	TV	IMP	YEAR	PRO	N	R^2^
(1) LCP	5.55*** (1.74)	-9.66*** (2.22)	1.56** (0.62)	0.00*** (0.00)	-0.00 (0.00)	0.90*** (0.24)	-0.00 (0.00)	control	control	372	0.84
(2) LCP	5.11*** (1.31)	-9.58*** (2.23)	1.77*** (0.64)	0.00*** (0.00)	0.00 (0.00)	0.93*** (0.29)	-0.00 (0.00)	control	control	341	0.81

Note: Cluster robust standard errors are in parentheses; ***, **, and *  represent significance at the 1%, 5%, and 10% levels, respectively

As can be seen from the table, the regression coefficient of the core explanatory variables lagged by one period is 5.55, which is significant at the 1% level; the regression coefficient of the core explanatory variables lagged by two periods is 5.11, which is significant at the 1% level. In summary, the regression results of lagging the core explanatory variables by one and two periods are consistent with the benchmark regression results, indicating that the results are robust.

#### 4.5.2. Instrumental variable approach.

Referring to the method of Baum [67] and other scholars adopt the 2SLS instrumental variable regression method for testing. Considering that instrumental variables need to satisfy the preconditions of exogeneity and correlation, this paper chooses the innovation performance (IP) as an instrumental variable to further test the relationship between regional innovation and entrepreneurship vitality and residents’ leisure consumption potential. By constructing the ivreg2 command in Stata, it is possible to report the F-statistic value of the Cragg-Donald test after the two-stage regression model [68]; the results are shown in Table 10.

**Table 10:** Endogeneity test with instrumental variables included.

Variable	(1)	(2)
	IEV	LCP
**IP**	0.002*** (0.001)	
**IEV**		48.5*** (15.84)
**MS**	−5.94** (2.63)	−5.94** (2.63)
**AYS**	−0.01 (0.02)	3.08*** (1.04)
**PBT**	−0.00 (0.00)	0.00*** (0.00)
**STC**	−0.00*** (0.00)	−0.00 * (0.00)
**TV**	0.00 (0.00)	0.92*** (0.23)
**IMP**	0.00 (0.00)	0.00 (0.00)
**YEAR**	control	control
**PRO**	control	control
**N**	403	403
**R** ^**2**^	0.21	0.49

Note: Cluster robust standard errors are in parentheses; ***, **, and *  represent significance at the 1%, 5%, and 10% levels, respectively.

The first-stage regression results show that the regression coefficient of the instrumental variable innovation performance (IP) on innovation and entrepreneurship vitality is 0.002, which is significant at the 1% level, indicating that the instrumental variable (IP) meets the correlation condition with the explanatory variable (IEV). After adding instrumental variables in the second stage, the regression between innovation and entrepreneurship vitality and leisure consumption potential is significantly positive and significant at the 1% level, indicating that the conclusion still holds after endogeneity issues are considered.

### 4.6. Mediation effect test

Table 11 shows the results of the mediation effect test of educational investment. Column (1) tests the direct effect of regional innovation and entrepreneurship vitality on leisure consumption potential, and the results show that innovation and entrepreneurship vitality has a significant positive effect on residents’ leisure consumption potential, with an *α*_*1*_ of 5.16. Column (2) tests regional innovation and entrepreneurship vitality on regional educational investment, and the results show that the higher the regional innovation and entrepreneurship vitality is, the greater the fiscal expenditure on education, with a *β*_*1*_ of 1322.4. Column (3) tests the indirect effect of innovation and entrepreneurship level on residents’ leisure consumption potential, and the results show that the indirect effect regression coefficient *γ*_*1*_ is 2.85 and the direct effect regression coefficient *γ*_*2*_ is 0.002.

**Table 11:** Mediation effect test of educational investment.

Variable	(1)	(2)	(3)
	LCP	EI	LCP
**IEV**	5.16** (2.42)	1322.4** (549.6)	2.815 (3.347)
**EI**			0.002** (0.001)
**MS**	−9.98*** (2.24)	−558.1*** (186.8)	−8.99*** (2.35)
**ATS**	1.63** (0.62)	−9.66 (39.45)	1.65** (0.62)
**PBT**	0.00*** (0.00)	0.006*** (0.002)	0.00*** (0.00)
**STC**	0.00 (0.00)	0.88** (0.32)	−0.001 (0.001)
**TV**	0.76*** (0.19)	8.62 (18.93)	0.75*** (0.18)
**IMP**	−0.00 (0.00)	0.00 * (0.00)	−0.00 (0.00)
**YEAR**	control	control	control
**PRO**	control	control	control
**N**	403	403	403
**R** ^**2**^	0.87	0.89	0.88

Note: Cluster robust standard errors are in parentheses; ***, **, and *  represent significance at the 1%, 5%, and 10% levels, respectively.

As can be seen from Table 11, the correlation between innovation and entrepreneurship vigor and leisure consumption potential is not significant after adding the mediating variable education input, indicating that education input plays a fully mediating effect in the process of regional innovation and entrepreneurship vigor affecting residents’ leisure consumption potential. The above analysis reveals that education investment plays a completely mediating role in the impact of regional innovation and entrepreneurship level on residents’ leisure consumption potential, which verifies Hypothesis 2.

### 4.7. Moderating effects test

Table 12 reports the results of the moderating effect test for science and technology investment (STI), column (1) reports the regression results after adding the moderating variable S&T investment, column (2) reports the regression results after adding S&T investment and the interaction term of IEV ×  STI. From column (1), the effect of the explanatory variable (IEV) becomes insignificant with the inclusion of the moderator variable (STI), suggesting that the direction and strength of the moderating effect offsets or masks the main effect. From column (2), the regression coefficients of the explanatory variables after adding the moderating variables and the interaction term are positive and significant at the 5% level, and the coefficient of the interaction term is negative and significant at the 5% level, which verifies Hypothesis 3.

**Table 12:** Test of moderating effects of scientific and technological investment.

Variable	(1)	(2)
	LCP	LCP
**IEV**	4.31 (2.99)	4.57** (2.17)
**STI**	0.001 (0.002)	0.007** (0.003)
**IEV×STI**		−0.011*** (0.004)
**MS**	−9.63*** (2.35)	−7.90*** (2.31)
**AYS**	1.63** (0.62)	1.74*** (0.60)
**PBT**	0.00*** (0.00)	0.00** (0.00)
**STC**	−0.000 (0.001)	0.00 (0.001)
**TV**	0.77*** (0.19)	0.79*** (0.20)
**IMP**	−0.00 (0.00)	−0.00 (0.00)
**YEAR**	control	control
**PRO**	control	control
**N**	403	403
**R** ^**2**^	0.87	0.88

Note: Cluster robust standard errors are in parentheses; ***, **, and *  represent significance at the 1%, 5%, and 10% levels, respectively.

The results show that with the increase in scientific and technological investment, the influence of innovation and entrepreneurship vitality on leisure consumption potential of residents is weakened. This may be because the target of science and technology investment is mainly enterprises, universities, research institutes, etc., which cannot cover the majority of resident, and the increase in fiscal expenditure on science and technology will lead to a relative decrease in fiscal expenditure for residents, which will lead to the situation of enterprises competing with people for profits [45] and reduce residents’ individual consumption motivation and willingness to spend on leisure consumption.

### 4.8 Heterogeneity analysis

#### 4.8.1 Policy heterogeneity.

The concept of “innovation and entrepreneurship” was formally introduced at the national level in 2013, and Table 13 reports the regression results before and after the 2013 policy change. Column (1) reports the regression results before the policy change, and the regression coefficient of innovation and entrepreneurship vitality on residents’ leisure consumption potential is −3.52, which is not significant (t = −0.38, p = 0.703). Column (2) reports the regression results after the policy change, and the regression coefficient of innovation and entrepreneurship vitality on residents’ leisure consumption potential is 6.59, which is significant at the 10% level (t = 4.59, p = 0.000).

**Table 13:** Heterogeneity test before and after the policy change.

Variable	(1)	(2)
	LCP	LCP
**IEV**	−3.52 (9.20)	6.59*** (1.33)
**MS**	−11.10 (8.892)	−9.24*** (1.40)
**AYS**	0.74 (0.67)	1.87*** (0.44)
**PBT**	0.00 (0.00)	0.00*** (0.00)
**STC**	0.002*** (0.001)	0.001 (0.001)
**TV**	−0.23 (0.19)	0.91*** (0.18)
**IMP**	−0.00 (0.00)	0.00 (0.00)
**YEAR**	control	control
**PRO**	control	control
**N**	93	310
**R** ^**2**^	0.99	0.99

Note: Cluster robust standard errors are in parentheses; ***, **, and *  represent significance at the 1%, 5%, and 10% levels, respectively.

The regression results show that after the concept of “innovation and entrepreneurship” was formally proposed at the national level in 2013, both enterprises and residents have paid attention to the importance of innovation and entrepreneurship as an indicator of personal ability, enterprise construction and social development, and people paid more attention to the cultivation of innovative thinking, expanding the demand for diversified consumption.

#### 4.8.2. Population structure heterogeneity.

The regional cultural environment plays an important role in promoting leisure consumption, and rich cultural connotations help to develop residents’ spiritual thoughts, thus further enhancing their willingness to consume leisure [69,70]. The plurality of regional cultures is influenced by the local demographic structure, and the inflow of outsiders drives cultural exchanges between different regions to a certain extent, thus contributing to the development of the local cultural environment [71,72]. In this study, the proportion of the external population to the total local population is used as a reflection of the regional demographic structure, and the average value of China’s proportion of outsiders (22.65%) is used to group the external population situation of 31 provinces and cities into high and low levels and to analyze heterogeneity.

In Table 14, Column (1) reports the regression results for the share of the external population below the mean, and the regression coefficient of innovation and entrepreneurship vitality (IEV) on the leisure consumption potential of the population is 8.81, significant at the 1% level; Column (2) reports the results of the regression of the external population share above the mean, and the regression coefficient of innovation and entrepreneurship vitality (IEV) on the potential to consume leisure is 3.09 and is not significant (t = 1.16, p = 0.248).

**Table 14:** Heterogeneity test for population structure.

Variable	(1)	(2)
	LCP	LCP
**IEV**	8.81*** (1.05)	3.09 (2.67)
**MS**	−2.89 (1.82)	−10.65*** (1.86)
**AYS**	1.73*** (0.45)	1.81*** (0.69)
**PBT**	0.00 (0.00)	0.00*** (0.00)
**STC**	−0.0003 (0.001)	0.0001 (0.0008)
**TV**	0.67*** (0.16)	0.79*** (0.14)
**IMP**	0.00 (0.00)	−0.00** (0.00)
**YEAR**	control	control
**PRO**	control	control
**N**	206	197
**R** ^**2**^	0.99	0.99

Note: Cluster robust standard errors are in parentheses; ***, **, and *  represent significance at the 1%, 5%, and 10% levels, respectively.

The regression results show that areas with a greater proportion of the external population help to unleash local leisure consumption potential, and cultural exchanges brought about by demographic diversity help to promote the enhancement of the local cultural environment, which, in turn, translates the fruits of innovation and entrepreneurship into the diversified consumption capacity of individual residents to a greater extent. The regression results show that regions with a lower percentage of external population are more significantly able to exert the role of regional innovation and entrepreneurial vitality in promoting residents’ leisure consumption. For regions with a relatively low proportion of external population, enhancing local innovation and entrepreneurship can boost the leisure consumption potential and help improve the slow development of local leisure consumption caused by the lack of external population.

## 5. Conclusion

This paper empirically analyzes the impact of regional innovation and entrepreneurship vitality on residents’ leisure consumption potential via using panel data from 31 provinces and cities in China from 2010–2022, and draws the following conclusions:

First, regional innovation and entrepreneurship vitality significantly promote residents’ leisure consumption potential, of which capital investment and technology level are the main dimensions that promote residents’ leisure consumption potential, and the number of entrepreneurs does not have a significant effect on the promotion of residents’ leisure consumption potential. Second, from the mediation effect test, innovation and entrepreneurship vitality can significantly increase the government’s financial investment in education, which in turn promotes a significant increase in residents’ leisure consumption potential. Third, from the moderating effect test, science and technology financial investment leads to the negative impact of regional innovation and entrepreneurship vitality on residents’ leisure consumption potential. Fourth, from the heterogeneity test, the impact of regional innovation and entrepreneurship vitality on residents’ leisure consumption potential will be significant for regions with fully supportive policies and larger external population.

On this basis, this paper proposes the following suggestions:

First, government departments should coordinate and cooperate to provide and guarantee the conditions and environment necessary for “mass innovation and entrepreneurship”, encourage and guide innovative and entrepreneurial behavior, and improve the quality of innovation and entrepreneurship. In terms of capital introduction, we should stimulate the investment vitality of social capital, shift from government direct investment to government-guided investment, and help startups overcome the problem of initial financing difficulties with the help of the government-guided funds for entrepreneurial investment [73]. In terms of technology, the introduction of foreign advanced technology should be digested, absorbed and reinnovated [74], and the development of independent core technology should be explored to promote the coordinated development of innovation and entrepreneurship.

Second, in terms of fiscal expenditure, the share of different contents of the expenditure should be reasonably distributed, and the function of financial expenditure on social resource redistribution should be fully considered. In terms of educational investment, it is necessary to close attention to the importance of fiscal expenditure on education for the cultivation of high-quality innovative talent, rationalize the arrangement and distribution of educational resources, provide effective educational resources to less developed regions [75], and avoid the emergence of the horse-trading effect of educational resources in different regions. In science and technology investment, timely supervision and implementation of science and technology financial investment in place, the secret profit-making behavior to be strictly investigated, to protect the purity and transparency of financial investment. In particular, against the backdrop of greater fiscal pressures on local governments, exploring the impact of fiscal inputs in different areas on the development of the digital economy and their mechanisms of action has a strong sense of urgency and importance in maximizing the effectiveness of fiscal funds.

Third, the advantage of a large domestic market should be fully utilized [76]. Given the complex and volatile international environment, the problem of overcapacity caused by the shrinking external market can be solved by maximizing the stimulation of domestic consumption demand [45]. In the new era, China takes high-quality development as the primary task of building a modern socialist country and must promote the development of new quality productive forces led by innovation and entrepreneurship on the supply side [14] and promote the transformation of the consumption structure by increasing the potential of leisure consumption of the residents on the demand side, so as to coordinate the promotion of supply-side structural reform and the transformation and upgrading of the demand side and to alleviate the contradiction between the need for a better life and the imbalance and insufficiency of development. sufficient development.

## 6. Contribution and limitations

The purpose of this paper is to study the inconsistency between economic development and spiritual needs in the context of the rapid development of the digital economy, which is based on the main contradiction in China, and to further explore the coordination between scientific and technological development and the needs of individuals for a better life.

First, the research and analysis idea integrates the transaction cost theory and evolutionary game theory, analyzes the relationship between enterprise innovation, government financial input and residents’ leisure consumption from the three levels of enterprise, government and residents, and develops the topic research in depth from the dynamic decision-making of multiple perspectives.

Second, the results of the study emphasize the necessity of sustained innovative behavior, and refute the paradox of a “smart crisis” [77]. Given that there are few studies on the relationship between regional innovation and entrepreneurship vitality and the leisure consumption potential of the population, this study adds to the literature in the fields of innovation and consumption and contributes to the dialectical understanding of the relationship between digital development and leisure consumption.

Finally, the results of the study help to better improve the efficiency of the use of government finances in the context of the digital economy. Unlike previous findings that finance promotes innovation and consumption, this study more concretizes and specializes the role played by government finance by distinguishing between different elements of finance. The findings of the study help to improve the efficiency of the use of government finances.

This study has certain limitations and provides potential possibilities for future research. Owing to the diversity of the content of financial investment, this paper only selects only part of the content of financial investment (education financial investment and science and technology financial investment) for research and analysis, and in the future, we can continue to enrich and expand the other content of financial investment to analyze the utility of government finance more comprehensively. In addition, this study is based on the trend of digitalization, innovation and entrepreneurship, which is a necessary condition for the development of the digital economy; however, innovation and entrepreneurship have increased, as the labor force structure of the problem is still worthy of attention, and this demographic change in the scope of the different impacts on the community needs to be further explored in depth.
